# Evaluation of breast cancer screening indicators in the female population using the National Health System, Brazil, 2018-2019: a descriptive study

**DOI:** 10.1590/S2237-96222023000200009

**Published:** 2023-05-08

**Authors:** Jeane Tomazelli, Maria Beatriz Kneipp Dias, Caroline Madalena Ribeiro, Mônica de Assis, Maria Asunción Sole Pla, Ellyete de Oliveira Canella, Arn Migowski

**Affiliations:** 1 Instituto Nacional de Câncer, Divisão de Pesquisa Populacional, Rio de Janeiro, RJ, Brazil; 2 Instituto Nacional de Câncer, Divisão de Detecção Precoce e Apoio à Organização de Rede, Rio de Janeiro, RJ, Brazil; 3 Hospital Quinta D’Or, Rio de Janeiro, RJ, Brazil

**Keywords:** Breast Neoplasms, Mass Screening, Brazilian National Health System, Indicators (Statistics), Population Studies in Public Health, Neoplasias de la Mama, Tamizaje Masivo, Sistema Único de Salud, Indicadores (Estadística), Estudios Poblacionales en Salud Pública, Neoplasias de Mama, Programas de Rastreamento, Sistema Único de Saúde, Indicadores (Estatística), Estudos Populacionais em Saúde Pública

## Abstract

**Objetive:**

to analyze breast cancer screening monitoring indicators in the female population using the Brazilian National Health System, from 2018 to 2019.

**Methods:**

this was a descriptive study based on Cancer Information System (SISCAN) data; screening indicators were calculated following deterministic linkage of the mammography and histopathology databases.

**Results:**

in 2018, 807,430 women aged 50 to 69 years were screened for breast cancer, 91% of whom had a benign result, 1.8% probably benign, 6.7% inconclusive results and 0.5% results suggestive of cancer; the positive mammogram rate was 9.0%; biopsy was estimated to be indicated for 1.6% of the women, 33.9% of whom had a malignant result, and the cancer confirmation rate was 5.4 per 1,000 women.

**Conclusion:**

high benign lesion loss to follow-up was identified; the positive mammogram rate was lower than the international parameter, but the cancer detection rate was adequate and the percentage of inconclusive mammograms was acceptable.

## Resumo

**Objetivo:**

analisar indicadores de monitoramento do rastreamento do câncer de mama na população feminina usuária do Sistema Único de Saúde, Brasil, no período 2018-2019.

**Métodos:**

estudo descritivo, a partir do Sistema de Informação do Câncer; foram calculados indicadores de rastreamento após relacionamento determinístico das bases de dados de mamografia e histopatologia.

**Resultados:**

em 2018, 807.430 mulheres na faixa etária de 50-69 anos foram rastreadas, e dessas, 91% apresentaram resultado benigno, 1,8% provavelmente benigno, 6,7% inconclusivo e 0,5% sugestivo de câncer; a taxa de mamografia positiva foi de 9,0%; estimou- -se indicação de biópsia para 1,6% das mulheres, resultado maligno para 33,9% delas, e taxa de confirmação de câncer de 5,4 por 1 mil mulheres.

**Conclusão:**

identificou-se elevada perda de seguimento das lesões benignas; a taxa de mamografias positivas foi inferior ao parâmetro internacional; contudo, a taxa de detecção de câncer foi adequada, e o percentual de mamografias inconclusivas, aceitável.

## Resumen

**Objetivo:**

analizar indicadores del tamizaje de cáncer de mama en población femenina atendida por Sistema Único de Salud Brasileño, de 2018-2019.

**Métodos:**

estudio descriptivo basado en Sistema de Información del Cáncer (Siscan). Los indicadores de detección se calcularon después de vinculación determinista de bases de datos de mamografía e histopatología.

**Resultados:**

en 2018, 807.430 mujeres de 50-69 años examinaram cáncer de mama. De estos, 91% tuvo resultado benigno, 1,8% probablemente benigno, 6,7% resultado no concluyente y 0,5% sugestivo de cáncer. La tasa de mamografías positivas fue 9,0%. Se estimó indicación de biopsia en 1,6% de las mujeres, resultado maligno en 33,9% y tasa de confirmación de cáncer de 5,4 por 1.000 mujeres.

**Conclusión:**

se identificó alta pérdida de seguimiento de las lesiones benignas. La tasa de mamografías positivas fue inferior al parámetro internacional, pero la tasa de detección de cáncer fue adecuada y el porcentaje de mamografías no concluyentes aceptable.

**Table:** 

Study contributions	
**Main results**	The BI-RADS^®^ categories 0, 4 and 5 (7.2%) and the cancer detection rate (5.4/1,000 women) found in mammogram screening in Brazil showed a pattern similar to that described in the literature, while the positive mammogram rate (9.0%) was lower.
**Implications for services**	Breast cancer screening indicators are influenced by data quality. As such, monitoring of mammography services, at different levels, should include monitoring of the quality of data reported by them.
**Perspectives**	The national analysis presented can serve as a reference for local analyses. Supported by indicators and quality criteria, identification of shortcomings and problems in breast cancer screening will help reduce its mortality.

## INTRODUCTION

Breast cancer is the most common type of cancer in the female population in Brazil, except for non-melanoma skin cancer. Estimates for the three-year period 2020-2022 point to 66,280 new breast cancer cases per year in Brazil, as well risk of 61.6 cases per 100,000 women.^1^ Between 1980 and 2018, breast cancer mortality increased 50.6%, revealing the challenge of controlling the disease nationwide.^2^

Some countries, such as England and Canada, have implemented organized screening programs, this being an early cancer detection strategy aimed at women without suspicious signs and symptoms.^3^ ^,^ ^4^ Breast cancer control action monitoring is carried out based on indicators, such as screening coverage, recall rates, altered mammography results, biopsy and cancer detection, which make it possible to follow up and evaluate the performance of these programs.^3^ ^,^ ^4^

In Brazil, early detection of breast cancer is a priority for the Ministry of Health;^5^ screening, however, is opportunistic, that is, women are screened when they spontaneously seek this health service.^6^

With effect from the implementation of the Breast Cancer Information System (Sistema de Informação do Câncer de Mama - SISMAMA) in the Brazilian National Health System (Sistema Único de Saúde - SUS) in 2009, the calculation of early breast cancer detection indicators was based on data related to examinations, given the absence of a unique identifier, such as the National Health Card (Cartão Nacional de Saúde - CNS) number, that would enable analysis of individualized data for monitoring the population. In 2013, with the implementation of the Cancer Information System (Sistema de Informação do Câncer -SISCAN), identification of service users undergoing examinations became possible through their CNS number, allowing better monitoring of the program’s actions.^7^

In Brazil, most of the studies published that analyze indicators of early breast cancer detection in the Brazilian population have been based on data related to mammogram results.^5^ ^,^ ^8^ ^,^ ^9^ The present study seeks to fill this gap by analyzing indicators obtained from the individual records of screened women registered on the SISCAN. Evaluation of the follow-up of this population may contribute to improved breast cancer monitoring and screening in Brazil.

The objective of this study was to analyze breast cancer screening monitoring indicators in the female population using the Brazilian National Health System, from 2018 to 2019.

## METHODS

A descriptive study was conducted to analyze indicators of breast cancer screening in the Brazilian female population, based on SISCAN data. The CNS number (unique identifier) was used to perform deterministic linkage of mammography and histopathology examinations held on the SISCAN databases and thus identify women screened in 2018, and those with subsequent examinations between 2018 and 2019.

In Brazil, breast cancer screening complies with the national guidelines established by the Brazilian Ministry of Health,^10^ which recommend the method, periodicity and age range for performing it. Mammography is the examination indicated to identify breast cancer in its early stages and must be registered on the SISCAN, when performed on the SUS.

The Ministry of Health recommends monitoring the indicators related to early detection of breast cancer using data held on the information systems. However, prior to public data held on SISCAN being made available in 2018, this analysis was limited to data derived from examinations and not from people examined.^5^

Mammography results are classified on the SISCAN according to the Breast Imaging Reporting and Data System (BI-RADS^®^), published by the American College of Radiology (ACR),^11^ and there are specific diagnostic investigation procedures for each BI-RADS^®^ category: BI-RADS^®^ categories 1 and 2 indicate the absence of lesions suspected of being malign, and women are advised to perform screening every two years, according to national guidelines;^10^ BI-RADS^®^ categories 0, 3, 4 and 5 are defined as abnormal results, demanding investigation by imaging examinations, radiological control, among other diagnostic procedures.^11^ ^,^ ^12^ In the case of mammograms with results falling in BI-RADS^®^ category 3, radiological control is recommended by means of a new mammogram within a period of six months to one year, while for those classified as BI-RADS^®^ 4 or 5, diagnostic investigation by means of biopsy and histopathology testing is recommended.

BI-RADS^®^ category 0 corresponds to a mammogram with an incomplete result, which requires comparison with previous examinations, additional mammography incidences and maneuvers, or breast ultrasound.^13^ Comparison with previous exams, new incidences and maneuvers must be performed by the radiology service before the definitive BI-RADS^®^ result is issued.^14^ As such, in accordance with what is recommended for this result, the SISCAN recommendation is to perform ultrasound.

For this study, we selected the records of female SUS service users aged 50 to 69 years who underwent breast cancer screening in 2018 and whose clinical indication for mammography was “screening in the target population”. Records having the following conditions were excluded: (i) anamnesis information indicating high risk of breast cancer; (ii) lumps larger than 20 mm in the radiology findings (considered clinically palpable lesions); (iii) record of previous mammogram results falling in BI-RADS^®^ categories 3, 4, 5 or 6 (taking results in 2017); and (iv) previous mammogram results falling in BI-RADS^®^ category 0, performed in 2017 or 2018 (considered to be a possible follow-up error).

In cases with more than one mammogram performed in 2018, we selected the examination with the most suspicious result, according to the following BI-RADS^®^ category order: 5, 4, 3, 2, 1 and 0. The BI-RADS^®^ result category 0 was defined as the last in this order, due to its being inconclusive and demanding diagnostic clarification by imaging and non-tissue methods.^11^

The results of histopathology breast tests recorded on the SISCAN in 2018 and 2019 were used to obtain information on diagnostic confirmation. We selected the records of female SUS service users, between 50 and 69 years of age, regarding biopsy and with anamnesis information reporting “detection by imaging”, i.e., non-palpable lesion identified by screening. Examinations classified as unsatisfactory were excluded. When there was more than one histopathology report with the same date, we selected the report with the highest severity, in the following order: positive for malignancy > suspicious core biopsy > indeterminate core biopsy > benign.

The study variables were:

- National Health Card (Cartão Nacional de Saúde - CNS) - unique numeric identification field;
- sex (female; male);
- age (in years);
- type of examination (mammogram; histopathology);
- clinical indication for mammography (screening; diagnosis);
- type of screening mammogram (target population; high risk population; person previously treated for breast cancer);
- high risk of breast cancer (yes; no; not known);
- lump (yes; no);
- lump size - descriptive numeric field;
- BI-RADS^®^ category - as per mammogram result: 0, 1, 2, 3, 4, 5 and 6;
- histopathology test result (benign; malignant); and
- date examination performed.

The mammography and histopathology examination data, including the CNS unique identification number, were obtained in the first quarter of 2020, corresponding to examinations recorded on the SISCAN between January 2018 and September 2019.

With the aim of ensuring the excellence of the examination results, we applied quality criteria when selecting the services that performed mammography and breast histopathology examinations. In the case of mammography, services that met the criteria defined by the researchers were included, namely:

- Performance of a number greater than or equal to 1,000 mammograms per year in 2018, this being an annual volume considered adequate to ensure the expertise of health professionals regarding their analysis of the images.^3^ ^,^ ^4^
- Performing mammograms for more than 500 women in 2018, with the aim of excluding services with high production resulting from repeat examinations in the same women.
- Having result distribution in accordance with acceptable parameters, as per the American College of Radiology, namely, (i) BI-RADS^®^ category 0 under 12% (desirable = 5% - 12%)^11^ and (ii) BI-RADS^®^ category 1 or 2 under 75%.^5^ ^,^ ^6^ ^,^ ^8^

With regard to laboratories that performed breast histopathology examinations, we selected those with annual production equal to or higher than 75 examinations/year, defined based on the median production observed. We considered that these services had greater scale and expertise in performing these examinations.

To avoid bias in the selection of the clinical indication and ensure that the first mammogram was a screening mammogram, we excluded women reported as being at high risk of breast cancer, those with previous examinations that indicated abnormality and those with clinically palpable lesions, according to the eligibility criteria described above.

In the case of women with screening mammograms in the BI-RADS^®^ 3 category, for which radiological control is recommended,^11^ we checked whether new mammograms had been recorded on the database provided. Mammograms performed at intervals of less than 60 days were not considered to be controls and were not included in the study. Since the recommendation for mammograms with results falling in BI-RADS^®^ category 0 presupposes assessment using ultrasound, we considered that all women with this result should have undergone breast ultrasound.

We calculated percentage distribution of screening mammogram results, follow-up mammogram results (BI-RADS^®^ category 3) and breast histopathology examination results. As breast ultrasound is not recorded on the SISCAN, the results obtained by Zanello et al.^15^, were used with the aim of estimating the distribution of ultrasound results for women with BI-RADS^®^ 0 screening mammography results. For this purpose, the percentages found in the ultrasound reports (46.1% for BI-RADS^®^ 1 or 2; 39.4% for BI-RADS^®^ 3; and 14.5% for BI-RADS^®^ 4 and 5)^15^ were multiplied by the percentage of women with a BI-RADS^®^ 0 screening mammography result in the present study.

The proportion of women undergoing biopsy following screening was estimated considering the percentages obtained in the following situations: (i) screening mammograms category 4 or 5; (ii) follow-up of BI-RADS^®^ category 3 when the subsequent mammogram result was BI-RADS^®^ 4 or 5; and (iii) follow-up of BI-RADS^®^ 0 mammograms when the ultrasound results were BI-RADS^®^ 4 or 5.

In this study we calculated the following indicators based on the results obtained: (i) percentage of BI-RADS 0, 4 and 5 mammograms, estimated by dividing the number of screened women with a BI-RADS^®^ 0, 4 or 5 result by the total number of screened women, multiplied by 100; (ii) the biopsy indication rate, estimated by the number of women with a BI-RADS^®^ 4 or 5 result divided by the total number of screened women in a year, multiplied by 100; (iii) the cancer detection rate, estimated by the number of cases of breast cancer detected in the screening divided by the total number of women screened in a year, multiplied by 1,000;^11^ and (iv) the positive mammogram rate, estimated by the number of screened women who required diagnostic investigation or control (BI-RADS^®^ 0, 3, 4 or 5) in relation to the total number of women screened in a year, multiplied by 100.^11^

We calculated the absolute and relative frequencies and the 95% confidence intervals (95%CI) of the BI-RADS^®^ categories. We used the R program (http://www.r-project.org), v.3.5.0 and its tidyverse package for database linkage and for the analyses.

The study project was approved by the Instituto Nacional de Câncer José Alencar Gomes da Silva Research Ethics Committee, as per Certificate of Submission for Ethical Appraisal No. 6944219.5.0000.5274.

## RESULTS

**Figure 1:**
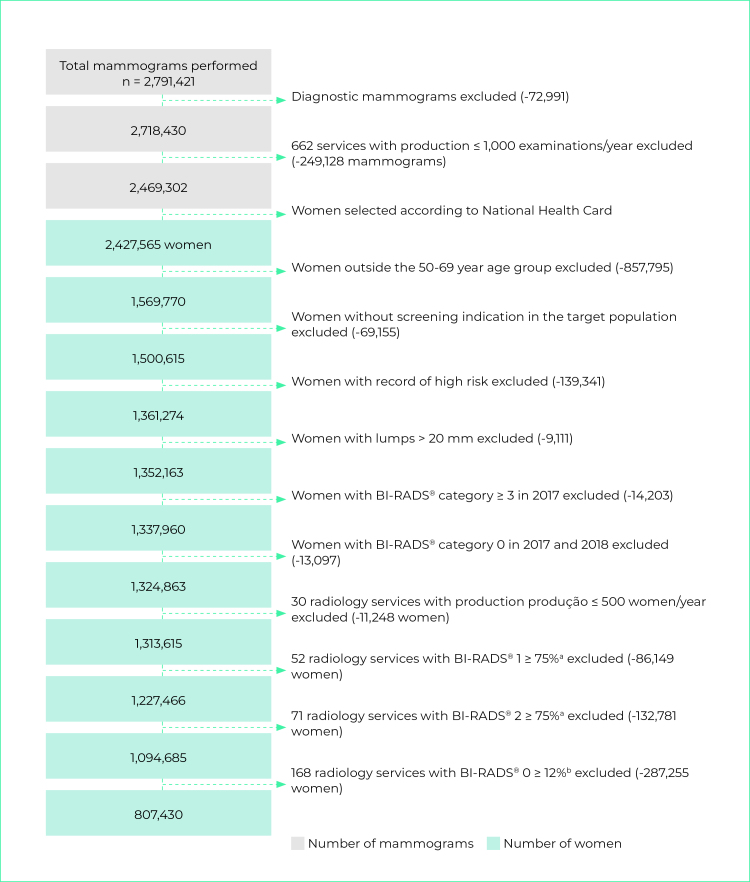
- Flowchart showing selection of females screened for breast cancer by the National Health System, Brazil, 2018

a) Services with over 75% of mammogram screening with BI-RADS^®^ category 1 or BI-RADS^®^ category 2: b) Services with over 12% of mammogram screening with BI-RADS^®^ category 0.

**Figure 2:**
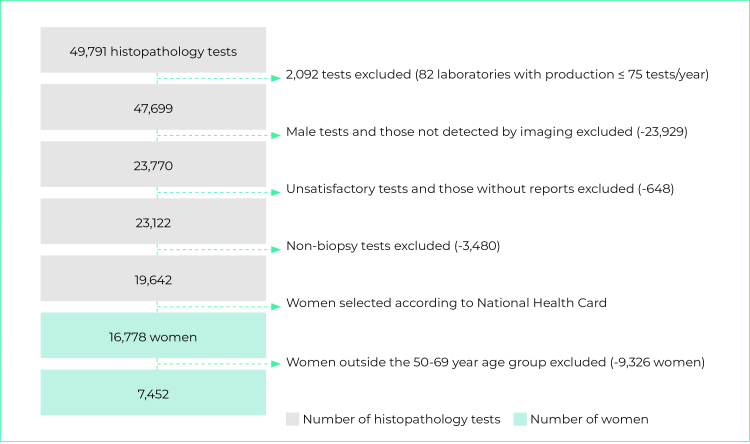
- Flowchart showing selection of records of females aged 50-69 who had pathology tests for breast cancer diagnosis on the National Health System, Brazil, 2018-2019

In 2018, 2,791,421 mammograms were recorded on the SISCAN. After applying the inclusion and exclusion criteria, we obtained a total of 807,430 female SUS service users aged 50-69, who underwent screening mammograms in 382 radiology services in Brazil (Figure 1). Between 2018 and 2019, 49,791 histopathology tests were recorded, from which we identified 7,452 female SUS service users also in the 50-69 age group, with tests performed by 110 laboratories (Figure 2).

**Figure 3:**
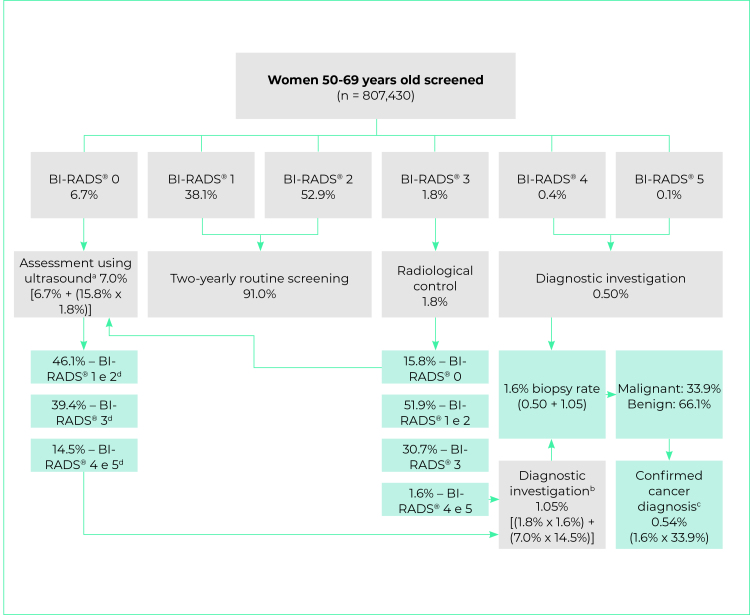
- Flowchart showing calculation of cancer screening indicators in females 50-69 years old using the National Health System, Brazil, 2018-2019

Of the 807,430 women screened in 2018, 91% had BI-RADS^®^ 1 or 2 results, for whom two-yearly routine screening was indicated; 1.8% had BI-RADS^®^ 3 results, for whom six-monthly or annual radiological control was indicated; 0.5% had BI-RADS^®^ 4 or 5 results, requiring diagnostic investigation with histopathology tests; and 6.7% had BI-RADS^®^ 0 results, requiring ultrasound (Figure 3). BI-RADS^®^ 0, 4 and 5 mammograms accounted for 7.2%; and the positive mammogram rate was 9.0%.

We identified 14,221 women with BI-RADS^®^ 3 mammography screening results; we found that 30.5% of them had radiological control on the SUS, with a new mammogram, as recommended by the current guidelines. The control mammography results were: 51.9% of women with unaltered mammograms or benign findings (BI-RADS^®^ 1 or 2); 30.7% new BI-RADS^®^ 3; 1.6% BI-RADS^®^ 4 or 5; and 15.8% BI-RADS^®^ 0 (Table 1).

**Table 1:** - Distribution of the results of BI-RADS^®^ category 3 breast screening and follow-up tests, in females 50-69 years old, performed by the National Health System, Brazil, 2018-2019

BI-RADS^®^ category	N	% (95%CI^a^)
Screening (n = 807,430)		
0	54,368	6.7 (6.7;6.8)
1	307,649	38.1 (38.0;38.2)
2	427,369	52.9 (52.8;53.0)
3	14,221	1.8 (1.7;1.8)
4	3,362	0.4 (0.4;0.4)
5	461	0.1 (0.1;0.1)
**Follow-up^b,c^ (n = 4,332)**		
0	683	15.8 (14.7;16.9)
1	745	17.2 (16.1; 18.3)
2	1,503	34.7 (33.3;36.1)
3	1,332	30.7 (29.4;32.1)
4	66	1.5 (1.2;1.9)
5	2	0.1 (0.0;0.1)
6	1	0.0 (0.0;0.1)

a) 95%CI: 95% confidence interval; b) Control mammogram; c) Considering only women in BI-RADS^®^ category 3 with information about new mammogram on the SISCAN.

Our analysis of the screened women found that after follow-up with ultrasound and mammography following initial screening in one year, 7.0% of women aged 50-69 needed ultrasound and 1.8% needed diagnostic mammography for radiological control.

Of the 7,452 women who had histopathology tests, 33.9% had malignant results and 66.1% had benign results. Taking the distribution of the mammogram results, ultrasound results, and diagnostic confirmation through histopathology tests, the biopsy indication rate estimated at 1.6%; and 0.5% of cases had diagnostic confirmation of cancer (Figure 3). The cancer detection rate was 5.4 cases per 1,000 screened women.

a) 6.7% BIRADS^®^ 0 mammogram screening added to 0.284% BIRADS^®^ 0 coming from BIRADS^®^ 3 mammogram screening radiological control (1.8% x 15.8%); b) 1.02% post-ultrasound BI-RADS^®^ 4 and 5 (7.0% x 14.5%) added to 0.03% BI-RADS^®^ 4 and 5 after BIRADS^®^ 3 radiological control (1.8% x 1.6%); c) 1.6% of women who needed biopsy multiplied by percentage of biopsies with malignant result (33.9%); d) Distribution of breast ultrasound results as per study by Zanello et al.^15^

## DISCUSSION

The analysis of the indicators showed that the rate of positive breast cancer screening mammograms in Brazil, 9.0%, was below the international parameter recommended by the American College of Radiology, while the biopsy indication rate (1.6%) and the cancer detection rate (5.4 cases/1,000 women screened) were close to the international benchmark values.^12^ ^,^ ^16^ ^-^ ^18^

BI-RADS^®^ 1 and 2 results were predominant and were close to the screening data (89.9%) presented in the 2019 ACR National Mammography Database (NMD) report,^16^ which includes women of all ages. The proportion of BI-RADS^®^ 0 results was within the acceptable parameter (5% to 12%),^11^ being lower than that presented in the NMD report (9.6%).^16^ Studies using SISMAMA data for Brazil as a whole and for Goiânia (capital of the state of Goiás) for the year 2010, indicate a predominance of BI-RADS^®^ 1 and 2 results and a proportion of examinations with BI-RADS^®^ 0 results close to the upper limit mentioned in the literature, which is above the proportion estimated in our study for women.^8^ ^,^ ^19^ In Brazil, a tendency can be seen in practice to replace mammography incidences and maneuvers with ultrasound, which could increase the occurrence of BI-RADS^®^ 0 in screening results. Although the SISMAMA data referred to examinations, they also referred to the SUS, and the comparison with the SISCAN data indicates that there is consistency, in part, between the results. It is worth noting that the present study excluded services with problems in the quality of information or mammography.

The proportion of BI-RADS^®^ 3 results found (1.8%) was lower than that found in 2011 in the state of Rio de Janeiro (3%);^20^ and higher than the ARC proportion (0.22%).^16^ The differences may be related to breast cancer incidence in the state of Rio de Janeiro being higher than the national average,^1^ as well as to the difference in age groups between the studies, and to the ACR recommendation not to use BI-RADS^®^ 3 assessment in the screening setting.^11^ To comply with the ACR recommendation, a complete diagnostic assessment is necessary in this case, reiterating the American Medicare health system guideline that the proportion of BI-RADS^®^ 3 screening mammograms should be close to zero.^11^

In Brazil as a whole, the proportion of BI-RADS^®^ 3 examination results was below 3% in 2010; ^8^ while in the state of Minas Gerais it was slightly above 3% in 2010, and close to 4% in 2011.^6^ A study conducted in a private institution in São Paulo covering the period 2010-2011, found that 8.3% of the results were in the BI-RADS^®^ 3 category in women with a mean age of 66 years.^21^ It is possible that studies using data from examinations without delimiting whether they relate to the same women, and without adopting data quality assessment criteria, may have artificially increased the proportion of BI-RADS^®^ 3 results, as may have studies that addressed older women.

The positive mammogram rate (11.6%) was lower than the ACR^11^ benchmark (10.6%) and that of the United States Breast Cancer Surveillance Consortium (BCSC), although the latter rate is defined from digital mammograms comprised of 60.9% of women aged 50-74 years, performed between 2007 and 2013.^12^ This indicator is more influenced by the proportion of BI-RADS® 0; therefore, the eligibility criteria in the present study, with a cutoff point for BI-RADS® 0, may partly explain this difference..

The Canadian breast cancer control program, which does not use the BI-RADS^®^ classification system, sets target abnormal result rates of less than 10% (first-time) and 5% (subsequent), and in 2011-2012, these rates were set at 15.3% for first-time mammograms and 7.2% for subsequent mammograms.^22^ Differences found among the various studies may be attributed to professional expertise and to the volume of tests services are required to perform.^23^ The change in the definition of positive mammograms in the versions of the ACR Atlas,^11^ ^,^ ^24^ need to be taken into consideration, in which previously only BI-RADS^®^ 0, 4 and 5 results were considered, and category 3 was excluded. Such differences may cause confusion in the comparison between studies. An example of this is the 12.1% recall rate due to abnormal results (BI-RADS^®^ 0, 4 and 5) in SUS services in the period 2010-2011.^5^

The total BI-RADS^®^ 4 and 5 results in screening mammograms, which indicate the need for biopsy, was lower than that found by the BCSC^12^ (1.71%) and by the São Paulo study^21^ (1.61%). The distribution of these categories in the NMD report^16^ in 2019 was 0.08%, including all ages. An important variation can be seen in these results.

Regarding follow-up of women, the proportion of BI-RADS^®^ 3 follow-up was close to that of the Rio de Janeiro study, performed with women screened in all age groups (29.5%), as well as being close to the distribution in almost all other categories - except BI-RADS^®^ 4, for which the proportion was higher.^20^ In the present study, almost 70% of women with BI-RADS^®^ 3 results were lost to follow-up, a phenomenon also identified in the Rio de Janeiro study.^20^ It is important to note that 1.6% of these women in radiological control later presented suspicion of malignancy that required histopathology examinations. This finding reinforces the need to devote special attention to women who have overcome the initial barrier of access to services but still need to ensure the continuity of their care.

The biopsy indication rate was close to that expected by the Irish program (≤ 2.0%),^17^ as well that recorded in the 2019 ACR report^16^ (1.66%), and that of Norwegian county programs (1.4%).^18^ Moreover, the rate is intermediate if one considers the Canadian results.^22^ According to the Canadian program, in 2011-2012, among women with altered mammography screening (15.3% screened for the first time and 7.2% subsequent screening), 14.9% had core biopsies and 1.7% had surgical biopsies, corresponding to 2.27% and 0.26%, respectively, of the need for biopsy. Therefore, 2.53% of women who underwent first mammogram screening underwent biopsy. As for the women who underwent subsequent screening, the biopsy rate was 1.2%, corresponding to 1.1% core biopsy and 0.1% surgical biopsy.

Local studies indicate different biopsy rates: 2.2% in South Africa;^25^ 1% in the municipality of Monteiro, state of Paraíba;^26^ and 1.7% in a reference center in Campinas, state of São Paulo, based on 35,041 mammograms.^14^ Age and type of mammography machine (digital or not) should be considered, as they influence the biopsy rate.^18^ There are also particularities in the studies and programs consulted. The biopsy rate estimated in the present study was generally close to those reported for national screening programs.

The cancer detection rate per 1,000 women screened was close to that of Norway^18^ (5.6), as well as to the parameters reported by the ACR^11^ (4.7), and to that found by the BCSC^12^ (5.1); however, it was lower than that of South Africa (10.0).^25^ When analyzing data from the United Kingdom National Health Service (NHS) breast cancer screening program, Burnside et al.^27^ also found a higher rate (8.1). In Canada, in 2011-2012, this rate was 4.9/1,000 women screened for the first time and 3.7 at subsequent screening, whereas rates of 5.0 and 3.0 respectively are predicted.^22^ In the United Kingdom, screening every three years may be one of the causes of the higher cancer detection rate there.

A study in Paraíba,^26^ with women aged 40 to 69 years, found a cancer detection rate of 3.4/1,000 screened women. In the São Paulo study, with women whose mean age was 66 years, a rate of 4.8 was found.^21^ The Campinas study cited above found a rate of 0.3%, possibly explained by the fact that 42.2% of the women included in the study were under 50 years of age and that the study only included 93% of subsequent screening.^14^ In general, despite the variability in BI-RADS^®^ 4 and 5 distribution, the detection rate estimated in our study was close to that reported in several programs and studies.

A possible limitation of the present study is the use of data from an information system with a heterogeneous degree of implementation nationwide, the gradual expansion of which reached mammogram coverage of around 74% by the SUS in 2019.^28^ On the other hand, the considerable volume of data and its national coverage are positive aspects, reducing the chance of the indicators calculated being distant from the real scenario in Brazil. Moreover, the unprecedented analysis of breast cancer screening indicators based on women’s records rather than examination records is an important move forward in relation to studies conducted in the country.^5^ ^,^ ^6^

The quality criteria used to select radiology clinics and laboratories aimed to mitigate shortcomings in the filling in of information about diagnostic conclusion, previously detected in SISMAMA data^19^ ^,^ ^29^ and in SUS radiology service assessments.^30^ If, on the one hand, these criteria may restrict the results to what would be expected in a scenario of quality screening tests - which does not happen uniformly in Brazil - on the other hand, as it is a population-based study, the results obtained provide parameters for evaluating and planning breast cancer screening actions. The eligibility criteria presented can also be useful for managers for monitoring services.

It is expected that the indicators presented will serve as a reference for future evaluative studies with longitudinal follow-up of women undergoing mammography, with quality criteria, contributing to moving forward with the organization of the breast cancer care line in Brazil.
